# Psychopathic Traits and Reactive-Proactive Aggression in a Large Community Sample of Polish Adolescents

**DOI:** 10.1007/s10578-013-0432-4

**Published:** 2013-12-28

**Authors:** Lidia Perenc, Mieczyslaw Radochonski

**Affiliations:** 1 Institute of Physiotherapy, Medical Faculty, University of Rzeszow, ul. Warszawska 26a, 35-215 Rzeszow, Poland; 2 Faculty of Education and Arts, University of Rzeszow, ul. Jalowego 24, 35-959 Rzeszow, Poland

**Keywords:** Adolescents, Psychopathic traits, Reactive-proactive aggression, Assessment

## Abstract

This paper presents results of the only large-scale study carried-out in Poland to date on the prevalence of psychopathic traits and their relationship with aggressive behaviour in mainstream adolescents. The sample consists of 9,415 students (4,808 boys, 4,607 girls) in the first to third grades at 142 public secondary schools. Psychopathic traits were measured by teacher-report ratings with the antisocial process screening device (APSD), while aggressive behaviours were assessed using the Reactive-Proactive Aggression Questionnaire. Analysis of results revealed that boys scored much higher than girls in total APSD scale measuring psychopathic traits. Only 2.68 % of assessed adolescents scored above the cut-off of 25 points. Results also showed significant correlations between psychopathic traits and both proactive and reactive aggression. The authors concluded that screening a large sample to identify children and youths with psychopathic traits has some important advantages but, on the other hand, it is a sensitive undertaking because of the label ‘psychopath’ can have negative consequences for the subjects.

## Introduction

During the last two decades in the professional literature there has been increasing interest in research on “psychopathic like traits” in children and adolescent and their association with aggression and antisocial behaviour. What is more, a growing number of mental health professionals believe that an early confirmation of the presence or absence of these features may help to identify unique etiological factors involved in the development of antisocial behaviour. This belief is based on a notion that the psychopathic traits are associated with the development and persistence of conduct disorders and antisocial behaviour in adolescents and adults. In this context, an early diagnosis may be very useful in predicting these kinds of problems in later life [1]. The concept of psychopathy has been proven useful for identifying severely violent and disruptive behaviour in adult criminals, especially recidivists [2]. Moreover, in the group of adolescent delinquents there is a subgroup that can be distinguished from others by the violence of their criminal acts, early onset of criminal behaviours and their repetitiveness [3]. Individuals representing this group are characterized by a set of personality traits such as, for example, manipulative tendencies, deceitfulness, lack of empathy and remorse, impulsiveness, and irresponsibility in relations with others. A number of studies have proved that the psychopathic constellation is composed of three main dimensions: (1) lying and manipulative interpersonal style (2) callous and non-empathetic emotional functioning (3) adventurous and irresponsible behavioural style [4]. The existence of three related sets of characteristics was confirmed by leading researchers in extensive studies performed with adult incarcerated criminals, both males and females [5, 6]. It is assumed that psychopathic traits are manifested at an early age, however, in clinical and empirical literature there is major criticism of the notion of “child and adolescent psychopathy”. Some authors suggest that it may be difficult to diagnose permanently distorted personality traits in children with sufficient certainty [7]. Those opinions emphasize the fact that a simple application of the psychopathy construct to children and adolescents is not risk free. They also stress that a wrong diagnosis may be harmful for a young individual in that it may label them for life. Nowadays, the debate about the assessment of psychopathic traits in young populations discussed in the literature covers two important topics: (1) to what extent are personality traits stable (2) is the psychopathy construct applicable to children and youth [8]. The answer to those questions is difficult because the stability of some traits ranges between moderate and high, while the stability of other, especially affective traits, appears moderate to low [9]. There are also serious concerns about the suitability of the instruments used in the assessment of young people with disturbed behaviour. For example, some authors argue that self-report scales used in assessment of psychopathic traits have little predictive value [10] or low internal consistency [11]. In the light of such opinions the question arises how should the “young psychopath” be identified? It is widely known, that the adult psychopaths are typically diagnosed with standardized tools such as the Hare Psychopathy Checklist-Revised [12]. The Hare Psychopathy Checklist-Youth Version has also been elaborated for use in adolescent population [13]. Among several measures used in the assessment of psychopathy in adolescents only two have been validated for screening in community settings: the Antisocial Process Screening Device-APSD [14] and the Youth Psychopathic Traits Inventory-YPI [15]. The screening for psychopathic-like traits in mainstream juvenile population seems to be very important for the identification of both severe and subclinical symptoms of the disorder. In many cases it may be crucial for the effective treatment and as well as for preventing those traits that foster an antisocial behaviour to become stable. Otherwise, those psychopathic individuals will become a substantial burden for society, including criminal justice system, educational institutions and mental health facilities. In terms of financial cost, for example, it has been estimated that in the USA preventing a single adolescent from becoming a recidivist saves American society more than $1.3 million [16].

## Aim of the Study

The present study is a contribution to the scientific literature on the prevalence of psychopathic-like traits in the general population of the secondary-school students. Very few studies exist that have reported findings about psychopathic traits in larger community samples [17] or that examined psychopathic features in large groups of young children and adolescents [18]. Further examination of these disorders is important for numerous reasons. First of all, identification of such traits may be helpful in the prediction of future aggressive behaviour of children and adolescents from the general population. Also in Poland, like in other countries, there are no normative samples that can provide an approximate prevalence of psychopathic traits [17], although only one-third of clinic-referred children have exhibited its high levels [19]. While rates in a non-referred community sample were expected to be low, the current study focused on the affective and narcissistic features that are typical for psychopathic disorders. Another important aim of this study was to estimate the relationship between psychopathic traits and aggressive behaviour in children. It was found in previous research that young people who possess psychopathic traits are more aggressive and antisocial in nature [20]. At the same time the literature concerning the relationship between psychopathic features and reactive and proactive aggression is inconsistent and has some contradictions.

There has been relatively little research on child and adolescent psychopathy in different ethnic and cultural groups. For example, some widely known authors, as Frick and Hare [14] argued that a great deal more research needs to be conducted to establish the utility of the instruments used in assessment of psychopathic traits (e.g. APSD) in different racial and ethnic groups. Such studies have been performed in several countries, both in Western and non-Western cultures, e.g. Fung et al. [21]. In Poland, like in other East European countries, this kind of research was not conducted yet. Even in the study on the prevalence and structure of psychopathic traits using a very large world sample (N = 33,016), Europe was represented only by German and Austrian populations, i.e. typical West European nations [22]. Our study attempts to fill this gap on ethnicity research by assessing psychopathic features in a very large community sample of adolescents from Poland (almost 10,000!). For example, in similar studies performed in other countries, relatively small populations were used (only several hundred participants).

Our first hypothesis was that prevalence of psychopathy-like traits in a large mainstream sample of Polish adolescents would not differ from results of similar studies performed in other countries. The second hypothesis was that adolescents with psychopathy-like traits show aggressive behaviours more often than their non-psychopathic peers. Our hypotheses are based on previous studies examining the relationship between psychopathy and aggressive behaviour. The results of such research support the assumption about the significant correlation between psychopathy features and aggression because both constructs pertain to difficulties in impulse control [23].

## Methods

### Study Population

This large-scale cross-sectional study was conducted with a 9,415 students from 142 secondary schools (junior high schools) located in the Podkarpacie province (south–east Poland). The schools were randomly selected from the total of over 500 secondary schools operating in Podkarpacie at time of the study. The participants made up 9.5 % of the total population of students attending junior high schools with total approximating 100,000 persons.

The sample represented relatively homogenous student population in terms of ethnicity, socioeconomic status, and religious affiliation (over 95 % are Polish and Roman Catholic). The age of students ranged between 13 and 16 years with mean of 14.38 years (SD = 2.1). Among 9,415 participants there were 4,607 girls (48.9 %) and 4,808 boys (51.1 %). The sociodemographic characteristics of the participants are presented in Table 1.

**Table 1:** Socio-demographic characteristics of participants

Age	13	14	15	16	Total
N	2,889	3,038	3,179	208	9,415
%	31.74	32.26	33.76	2.24	100.00
Gender	Girls		Boys		Total
N	4,607		4,808		9,415
%	48.93		51.06		100.00
Social milieu	Urban		Rural		Total
N	3,994		5,421		9,415
%	57.57		42.43		100.00
Class (grade)	Ist	IInd	IIIrd		Total
N	3,076	3,023	3,314		9,415
%	32.67	32.11	35.22		100.00

### Measures

#### Antisocial Process Screening Device (APSD)

The APSD is a 20-item instrument designed to measure antisocial behaviour in children and younger adolescents. The APSD was based on the Psychopathy Checklist-Revised, a measure designed for the assessment of psychopathic traits in adults (12-Hare, 2003). The items belonging to APSD assess three different dimensions: (1) callous/unemotional (CU) (2) narcissism (N) (3) impulsivity (I). High scores on these scales indicate high intensity for each particular trait. The items are graded on a 3-point scale: 0—not at all true, 1—sometimes true, 2—definitely true. Among twenty APSD items five score inversely (2, 7, 12, 18 and 20). The APSD can be completed by the child’s parent or teacher. There is also a child self-report version of the APSD which was used in several studies performed in the past. The results supported the 3-factor structure of the device in this format [24]. In the USA the APSD was normalized in a population of 1,120 school children whose mean age was 10.1 years. The results of statistical analyses showed a satisfactory level of internal consistency [14]. Also the past studies with older children and adolescents had suggested that teacher-report version used in assessment of psychopathic traits is significantly more valid than parent-report one [25]. This supports the assumption that the APSD can be used as a reliable measure of psychopathic traits in younger adolescents at the school settings. Internal consistency reported for the APSD in a community sample has been adequate for total score (.78–.81), but has been less desirable for factor scores, ranging from .50 to .68 [11]. Nevertheless, the APSD is the most widely used and researched brief psychopathy measure in the United States [26, 27].

There is still no standard cut-off score recommended for the APSD which would allow for categorical comparisons of psychopathic traits and classification of participants into rating high and low. However, some researchers use a cut-off score of 20 [28], while others use a cut-off score of 25 [29]. Also some researchers use a median-split [30]. In this study the cut-off score of 25 was used to indicate high intensity of psychopathic traits. For theoretical reasons, in addition to using the total APSD score, researchers often use the callous-unemotional subscale score to distinguish a sub-sample of high-scoring children who have been shown to be more severe and persistent in their psychopathic behaviour compared with low scorers on the callous-unemotional subscale. We therefore use both the total APSD and the callous-unemotional subscale score for analyses. Binary variables (high/low) were created by grouping children into two groups using cut-off score of 25.

Rather than imposing a categorical distribution on the sample, the whole range of the APSD was used in the present study in order to retain as much information from the scale as possible. In our study the Polish translation of the original instrument was used. The English version of the questionnaire was adapted and translated according to guidelines that are widely accepted for the successful translation of instruments in cross-cultural research [31]. As proposed by Brislin [31], one bilingual translator who was also a native speaker or a culturally informed individual blindly translated the questionnaires from the original language (English) to the second language (Polish) and another bilingual translated it back to the original language (Polish back to English). Differences in the original and the back-translated versions were discussed and resolved by joint agreement of both translators. This procedure was also applied to the second instrument used in this study.

#### The Reactive-Proactive Aggression Questionnaire (RPAQ)

The RPAQ [32] is a 23-item paper-and-pencil measure designed to assess proactive and reactive aggression. Reactive aggression (hostile or impulsive aggression) refers to angry and impulsive responses, which are usually triggered by provocation or stimuli subjectively interpreted as provocation. Proactive aggression (instrumental or premeditated aggression) refers to goal-oriented (e.g., bullying, stealing money), predatory aggression which is typically unprovoked. The Proactive Aggression scale is comprised of 12 items (e.g., had fights with others to show who was on top) and the Reactive Aggression scale includes 11 items (e.g., gotten angry when others threatened him). Each item is rated as 0 (never), 1 (sometimes), or 2 (often). The items reflect physical or verbal aggression. The Proactive, Reactive, and Total Aggression scales have all demonstrated alphas above .80 [32]. Raine et al. [32] reported internal reliabilities in excess of .83 for all three scales across two samples. The RPAQ has also demonstrated good criterion, convergent and discriminant validity. Confirmatory factor analysis of the RPAQ supports a two-factor, reactive/proactive model [32, 33]. In this study aggressive behaviour was rated by the students’ teachers.

A basic socio-demographic data related to the participants were taken from the school records.

### Procedure

This was a cross-sectional study, and it investigated the subjects’ selective personality traits and behaviours retrospectively. In order to conduct this assessment, co-operation was requested from teachers of secondary schools involved in this study. At first a formal approval was given by the education administration authority (Kuratorium Oswiaty) from the Podkarpacie province. After that, the permission to conduct the study was obtained from principals of all selected schools and parents’ committees. All parents provided informed consent and the children provided their assent. The people in charge of the data collection tasks were teachers tutoring the group of students belonging to the class randomly selected to the study. They knew the child best and therefore they completed a questionnaires to assess children’s psychopathic traits, and aggressive behaviour. The nominated teachers were initially trained during a special meetings organized in different locations where they learned how to administer the measures. Both assessment tools were used with each student and the socio-demographic data were collected completely anonymously. Children, whom the teacher knew less than one school-term, were excluded from the study. Copies of the teacher version of the scales were taken by the researchers for purposes of the quantitative analysis. The data collection occurred at schools after structured educational time. The teachers were not compensated financially.

This study was conducted in accordance with the ethical code being in operation at the University of Rzeszow.

## Results

The present study examined the prevalence of clinically significant psychopathic traits in the population of junior high-school students and the relationship between psychopathic features and reactive/proactive aggression. Statistical analysis of results was performed using SPSS version 18.0.

Table 2 reports the means and standard deviations of the main study variables and their correlations with gender and age of participants. Importantly, in this study the level and distribution of psychopathic traits, as measured by the teacher-report on the APSD, was relatively low. That is, the mean and standard deviation of the total APSD score in the Polish sample (mean = 6.52; SD = 3.73) is much lower than the score reported for the normative sample (mean = 9.71; SD = 8.22) [14]. Results indicated that boys had higher levels of callous-unemotional trait (M = 3.38), narcissism (M = 1.44), and impulsivity (M = 2.21) than girls (M = 3.05; 1.15 and 1.86 respectively). There are some associations between psychopathic-like traits and gender and age of the examined adolescents. Firstly, gender was consistently associated with proactive aggression. Boys were rated by teachers as performing more acts of proactive aggression than girls. However, there was a much smaller difference between the two genders in relation to reactive aggression. In general, age was significantly associated with reactive aggression. The teachers reported that older students usually indicated higher levels of proactive aggression than younger ones. For teacher-reported psychopathic traits, total APSD score remained significantly associated both with gender and age. In boys total ASPD score (M = 7.03) was much higher than in girls (M = 6.06). Adolescents at age of 15 years had the highest scores in total APSD scale (M = 7.22). The association between the subscale scores and socio-demographic variables varied. The CU scores were significantly correlated with age. According to teacher assessment the older students were presenting more distinct symptoms of callous-unemotional traits. The impulsivity subscale score showed stronger association with gender than age. Boys usually scored higher than girls. It should be noted that the differences between two genders in the mean levels of psychopathic traits were not statistically tested. This is why a care should be taken when interpreting these differences.

**Table 2:** Main study variables and sociodemographic traits

Psychopathic traits (N = 9,415)	Mean	SD	Gender		Age			
			Boys^a^	Girls^b^	13	14	15	16
Callous/unemotional	3.21	1.28	3.38	3.05	2.86	3.12	3.61	3.25
Narcissism	1.28	.65	1.44	1.15	1.16	1.19	1.42	1.33
Impulsivity	2.03	.74	2.21	1.86	1.92	1.97	2.19	1.90
Total APSD score	6.52	3.73	7.03	6.06	5.94	6.28	7.22	6.48
Proactive aggression	2.67	1.46	2.95	2.38	2.49	2.76	2.78	2.65
Reactive aggression	7.06	3.15	6.99	7.13	6.91	7.12	7.28	6.88

*SD* standard deviation

^a^N = 4,808, ^b^ N = 4,607

As it is shown in Table 3, there are some significant correlations between psychopathic traits and aggression. In all cases measures of both proactive and reactive aggression were positively and significantly correlated with particular measures of psychopathic traits. The strongest association occurred between the total APSD and proactive aggression scores (r = .375), and between total aggression and total APSD scores (r = .354). The total scores of 25 points helped to distinguish between low and high intensity of psychopathic traits in assessed adolescents. In our study the APSD appears to assess mostly the behavioural features of psychopathy. Nevertheless, it also comprises, with some validity, interpersonal and affective features of the disorder.

**Table 3:** Correlation between psychopathy traits and aggression

Psychopathic traits	PA	RA	Total
CU	.269*	.184*	.216*
N	.295*	.273*	.245*
I	.312*	.356**	.281**
Total APSD score	.375**	.308**	.354**

*PA* proactive aggression, *RA* reactive aggression, *CU* Callous/unemotional, *N* narcissism, *I* impulsivity

* *p* < .05; ** *p* < .01

As indicated in Table 4, only 253 adolescents (i.e. 2.68 % of examined population) displayed clinically significant traits of the psychopathic disorder. According to the teacher reported assessment there is a substantial difference between both genders in prevalence of high intensity of psychopathic traits. Only 83 girls (1.75 %) scored at or above 25 points in the total APSD scale. Using the same cut-off score it appeared that twice as many boys (170; 3.53 %) showed a similar level of psychopathy. This finding is in accordance with results of the studies in adult populations, which indicates that psychopathy is more prevalent in males than in females [34]. In several other studies involving different psychopathy scales males scored higher than females [35].

**Table 4:** Clinically significant scores on teacher-reported APSD subscales and total scores (numbers and percentages of participants by subscale)

APSD	Above cut-off score				Below cut-off score			
	Boys		Girls		Boys		Girls	
	N	%	N	%	N	%	N	%
CU	268	5.58	107	2.31	4,540	94.42	4,500	97.69
N	335	6.96	180	3.91	4,473	93.04	4,427	96.09
I	714	14.8	342	7.42	4,094	85.15	4,265	92.58
Total score	170	3.53	83	1.75	4,638	96.47	4,524	98.25

*CU* callous/unemotional, *N* narcissism, *I* impulsivity

## Discussion

The professional literature offers a number of empirical data supporting a relationship between psychopathy and different forms of aggression in the adult population [36]. However, this kind of relationship has not been well established in child and adolescent groups. Reliable findings suggest that callous-unemotional features are correlated with both reactive and proactive aggression [17].

Findings of our research are consistent with other studies suggesting that proactive and reactive aggression are uniquely associated with psychopathy and other forms of psychopathology. For example, Fite, Stoppelbein and Greening [37] have found that high levels of proactive aggression were associated with high levels of callous-unemotional traits, while reactive aggression was uniquely positively related to depressive symptoms and suicidal behavior and unrelated to behavioral consequences and callous-unemotional traits. In similar study performed by Marsee and Frick [38] it was showed that psychopathic traits are associated with higher rates of aggression and, with some limitations, delinquency in youth. The utility of psychopathic trait ratings was most evident for predicting ratings of aggression, in which both self-report and teacher-report of psychopathic traits were associated with overt and relational aggression. Using a similar methodology, Barry et al. [39] examined the role of psychopathy-linked narcissism in predicting proactive and reactive aggression and conduct problems in a group of moderately to highly aggressive children. Consistent with the study’s hypotheses, narcissism predicted unique variance in both proactive and reactive aggression, even when controlling for other dimensions of psychopathy, demographic variables associated with narcissism, and the alternative subtype of aggression. As hypothesized, impulsivity was significantly associated with only reactive aggression. Callous-unemotional traits were not related to proactive or reactive aggression once the control variables were entered. All dimensions of psychopathy predicted unique variance in conduct problems.

Psychopathic factors such as narcissism and impulsivity may be considered significant predictors of aggression. The fact that approximately 2.5 % of the adolescents from south to eastern Poland present significantly distinct features of psychopathy seems to be of great importance. For example, Forth and Burke [40] found features of psychopathy in 3.5 % of young people in community care, 12 % in those on probation and 28.3 % in those incarcerated. Our findings provide strong support for the continuation of similar studies not only in different parts of Poland but also in other East-European countries. The results of this study support the assumption tested in many past studies about association between psychopathic traits and higher rates of aggression. The practical utility of psychopathic trait ratings in the general population of adolescents seems to be obvious. As it is widely known, the assessment of psychopathic traits in children and adolescents is still in its stage of infancy. The findings of this study add some contribution to the international literature suggesting that juvenile psychopathy shows similar features to adult psychopathy (e.g. association with aggression and antisocial behaviour). As it was evidenced [17, 41], psychopathic traits are reasonably stable in adolescence. Thus there is a need for screening its prevalence in general population of adolescents. Also the juvenile psychopathy is an important predictor of educational and institutional outcomes. Early diagnosis of psychopathic traits may be helpful in planning more effective strategies aimed at prevention and treatment of behavioural disorders in children and youth. It is especially important nowadays when juvenile conduct problems, criminality and violence continue to present a great challenge in many countries, including Poland. It should be noted that most of the hitherto research on psychopathic traits in adolescents was conducted with incarcerated samples, while only some studies have focused on community-based adolescents. The extension of the research into these ‘‘normal’’ populations is essential for distinguishing normative adolescent traits from those more pathological with the “flavour” of psychopathy. Also, it is important for the future that longitudinal studies should be conducted on the developmental progression of psychopathic traits across childhood and adolescence into adulthood.

This study should be considered within its limitations as one of the first attempts of large scale screening of psychopathic traits in subjects entering the adolescence period. In Poland the youngsters attending junior high schools have a reputation of trouble-makers. Also this study relies on teacher informant ratings as a means of identifying the subjects presenting psychopathic traits and aggressive behaviours. Reliance on one informant in this study possibly excludes important information from other sources, such as parents and colleagues, who see the child’s behaviour from different perspectives. Additionally, the results obtained within this methodological approach potentially inflate the association between variables.

## Summary

The prevalence of psychopathic traits in examined population was 2.68 % (3.53 % boys, 1.75 % girls). The results of our research evidence that there is a reasonable correlation between juvenile psychopathic traits and aggression. Screening of psychopathic traits in adolescents has some important benefits, as, for example, early identification of high-risk offenders, reducing negative effects of labelling, and improving treatment effectiveness. More research is needed on different cut-off point values related to the instruments used in screening for psychopathic traits in adolescent cohorts, and mental health professionals should decide about the level of scoring required for recognition a high risk of psychopathy among adolescents.

Because of some overlap in symptomatology between psychopathy and aggression, future measures of psychopathic traits among adolescents should take into account the variability in behaviour over time and its socio-cultural context. Despite its obvious limitations, this research makes an important contribution in being the first large-scale study carried out in Poland on the prevalence of psychopathic traits in mainstream adolescents. It would be important to replicate this study with a large community sample in other East European countries as, for example, Ukraine and Slovakia. Such epidemiological studies might also offer a new concepts of the disorder and the factors that moderate its expression and development.
